# Traffic Density Exposure, Oxidative Stress Biomarkers and Plasma Metabolomics in a Population-Based Sample: The Hortega Study

**DOI:** 10.3390/antiox12122122

**Published:** 2023-12-15

**Authors:** Laura Sanchez-Rodriguez, Marta Galvez-Fernandez, Ayelén Rojas-Benedicto, Arce Domingo-Relloso, Nuria Amigo, Josep Redon, Daniel Monleon, Guillermo Saez, Maria Tellez-Plaza, Juan Carlos Martin-Escudero, Rebeca Ramis

**Affiliations:** 1 Integrative Epidemiology Group, Department of Chronic Diseases Epidemiology, National Center for Epidemiology, Instituto de Salud Carlos III, 28029 Madrid, Spain; laura.sanchez@isciii.es (L.S.-R.); ad3531@cumc.columbia.edu (A.D.-R.); rramis@isciii.es (R.R.); 2 Joint Research Institute-National School of Health (IMIENS), National Distance Education University, 28029 Madrid, Spain; 3 Department of Communicable Diseases, National Center for Epidemiology, Instituto de Salud Carlos III, 28029 Madrid, Spain; 4 CIBER on Epidemiology and Public Health, Instituto de Salud Carlos III, 28029 Madrid, Spain; 5 Department of Biostatistics, Mailman School of Public Health, Columbia University, New York, NY 10032, USA; 6 Biosfer Teslab, 43201 Reus, Spain; namigo@biosferteslab.com; 7 Department of Basic Medical Sciences, Universidad de Rovira i Virgili, 43007 Tarragona, Spain; 8 Institute for Biomedical Research, Hospital Clinic de Valencia (INCLIVA), 46010 Valencia, Spain; 9 Department of Biochemistry and Molecular Biology, Faculty of Medicine and Dentistry, Clinical Analysis Service, Hospital Universitario Dr. Peset-FISABIO, Universitat de Valencia, 46020 Valencia, Spain; guillermo.saez@uv.es; 10 Department of Internal Medicine, Hospital Universitario Rio Hortega, University of Valladolid, 47012 Valladolid, Spain; juancarlos.martinescudero@uva.es

**Keywords:** traffic density, metabolomics, air pollution, population-based

## Abstract

Exposure to traffic-related air pollution (TRAP) generates oxidative stress, with downstream effects at the metabolic level. Human studies of traffic density and metabolomic markers, however, are rare. The main objective of this study was to evaluate the cross-sectional association between traffic density in the street of residence with oxidative stress and metabolomic profiles measured in a population-based sample from Spain. We also explored in silico the potential biological implications of the findings. Secondarily, we assessed the contribution of oxidative stress to the association between exposure to traffic density and variation in plasma metabolite levels. Traffic density was defined as the average daily traffic volume over an entire year within a buffer of 50 m around the participants’ residence. Plasma metabolomic profiles and urine oxidative stress biomarkers were measured in samples from 1181 Hortega Study participants by nuclear magnetic resonance spectroscopy and high-performance liquid chromatography, respectively. Traffic density was associated with 7 (out of 49) plasma metabolites, including amino acids, fatty acids, products of bacterial and energy metabolism and fluid balance metabolites. Regarding urine oxidative stress biomarkers, traffic associations were positive for GSSG/GSH% and negative for MDA. A total of 12 KEGG pathways were linked to traffic-related metabolites. In a protein network from genes included in over-represented pathways and 63 redox-related candidate genes, we observed relevant proteins from the glutathione cycle. GSSG/GSH% and MDA accounted for 14.6% and 12.2% of changes in isobutyrate and the CH_2_CH_2_CO fatty acid moiety, respectively, which is attributable to traffic exposure. At the population level, exposure to traffic density was associated with specific urine oxidative stress and plasma metabolites. Although our results support a role of oxidative stress as a biological intermediary of traffic-related metabolic alterations, with potential implications for the co-bacterial and lipid metabolism, additional mechanistic and prospective studies are needed to confirm our findings.

## 1. Introduction

Traffic-related air pollution (TRAP) from motorized vehicles (passenger cars, motorbikes, heavy-duty vehicles) is a major source of air pollutant constituents, such as nitrogen oxides (NO_x_) and primary particulate matter (PM_2.5_), including black carbon [1,2,3]. TRAP has been associated with several detrimental health outcomes, including asthma onset and mortality endpoints (circulatory, ischemic heart disease and lung cancer) [4]. Some studies have reported a potential link of exposure to air pollution with alterations in specific metabolic pathways, including amino acids, purines, lipids and redox-related pathways [5,6]. In mechanistic studies, TRAP exposure consistently causes damage at the molecular level as well, such as generating reactive oxygen species (ROS) and directly altering the levels of metabolites such as fatty acids, amino acids and others, including glycine, serine, alanine and threonine and metabolites from the glycolysis and gluconeogenesis cycles [7,8]. Moreover, the presence of ROS in an organism can independently lead to additional metabolic alterations [9,10,11].

However, the negative health impacts of road traffic are not only attributable to exposure to air pollutants. Road-related traffic noise and the absence of green and blue spaces have been associated with metabolically unhealthy lifestyles (less physical activity, obesity) and an increased risk of certain diseases, such as type 2 diabetes [12,13,14]. Thus, population-based mechanistic studies on potential metabolic and redox effects from integrative traffic intensity measures are needed.

The main objective of this study was to evaluate the cross-sectional association between traffic density on the street of residence and urine oxidative stress biomarkers and plasma metabolomic profiles. We were also interested in exploring the potential biological implications of the findings through an in silico bioinformatic analysis (over-representation and network analysis of relevant metabolite pathways and redox-related candidate genes). Secondarily, we assessed whether oxidative stress could explain the association between exposure to traffic and plasma metabolite levels (i.e., the amount of change in traffic-associated metabolite levels that can be attributed to oxidative stress).

## 2. Methodology

### 2.1. Study Population

The Hortega Study cohort is a representative sample of the general population of Valladolid, Spain, obtained through a multi-stage complex sampling study. The study population consisted of beneficiaries from the universal public health system corresponding to the catchment area of the University Hospital Rio Hortega (Valladolid, Spain). In 2001–2003, the Hortega Study participants were examined and interviewed and provided biological samples. Details of the study design and data collection have been previously reported [15]. Among the 1502 recruited participants, we excluded 310 participants with insufficient plasma sample for metabolomic determinations, 40 participants missing BMI, 11 participants missing urine cotinine, 6 participants missing tobacco smoking variables, 3 participants missing education level and 2 participants missing oxidative stress markers, leaving 1181 subjects for the final analysis. The Ethics Committee of the Rio Hortega University Hospital approved the research protocol, and every participant provided informed consent.

### 2.2. Traffic Density

To assess the effect of traffic exposure on oxidative stress and metabolite levels, we used the traffic density of the closest roads to the home address. For this, we first merged the Navteq cartography with the official cartography from the Ministry of Transport, Mobility and Urban Agenda [16], which provides data on the total volume of vehicles circulating every Spanish road or street. We subsequently estimated individual exposure to traffic in the residence road by creating a 50 m buffer around the geographic coordinates of each participant’s home address. Finally, we computed the traffic density within the buffer as the total number of vehicles passing through the buffer over a year divided into 365.25 days (continuous variable expressed in vehicles per day) [17], which is a proxy reflecting how active a road is. For the descriptive analysis, we categorized traffic density into three groups using tertiles (<20.71 (low), 20.71–45.71 (moderate) and >45.71 (high)) cars/day.

### 2.3. Plasma Metabolite Levels

Metabolomic profile was determined by nuclear magnetic resonance (NMR) spectroscopy in non-fasting plasma. An amount of 82 µL of D_2_O was added to 418 µL of blood plasma and placed in a 5 mm NMR tube. NMR spectra were recorded using a Bruker Avance DRX 600 spectrometer (Bruker GmbH, Berlin, Germany). A single-pulse pre-saturation experiment was conducted in all samples, which were kept at 37 °C. To reference the spectra, the doublet of alanine at 1478 ppm was used. To eliminate differences in metabolite total concentration, the spectra were binned into 0.01 buckets and normalized to total aliphatic spectral area. Signals belonging to selected metabolites were quantified using semi-automated in-house MATLAB 6.5 (The MathWorks Inc., Natick, MA, USA) integration and peak-fitting routines. Chenomx NMR Suite V.4.5 software and two-dimensional (2D) NMR methods including homonuclear correlation spectroscopy and heteronuclear single-quantum correlation spectroscopy were used to identify and subsequently confirm the results [18].

In addition, an extended lipoprotein profile was assessed using the Liposcale^®^ methods for NMR spectra analysis. An amount of 500 μL of blood plasma samples was shipped on dry ice to the Biosfer Teslab (Reus, Spain) to determine lipoprotein lipid composition, size and the particle concentration of their respective subclasses (large, medium and small). Particle concentrations and lipoprotein subtypes were determined using the distinctive signals of the lipid methyl group. The size of a given subtype was evaluated by its diffusion coefficient. Common conversion factors were used to convert concentration units into volume units. The particle numbers of each lipoprotein subtype were estimated dividing the lipid volume by the particle volume of a given class.

All metabolites were adjusted by fasting time (hours) using linear regression. We then recalibrated the distribution of resulting metabolite residuals to metabolite-specific mean levels observed in the subset of individuals reporting fasting condition.

### 2.4. Oxidative Stress Biomarkers

The percentage ratio of oxidized (GSSG) to reduced (GSH) glutathione (GSSG/GSH%) and malondialdehyde (MDA) and the presence of the damaged base 8-oxo-7,8-dihydro-2′-deoxy-guanine (8-oxo-dG) were measured in urine. Analysis of GSSG and GSH levels was performed using high-performance liquid chromatography (HPLC) [19,20]. Additionally, MDA was quantified through spectrophotometric measurement at 532 nm following the MDA-thiobarbituric acid method [21]. Detection of 8-oxo-dG was achieved using high-performance liquid chromatography with electrochemical detection (HPLC-EC) [22,23]. To account for urine dilution, oxidative stress biomarker data were divided by urine creatinine levels and reported in nanomoles per millimole of creatinine. The measurement of urine creatinine was carried out using the modified kinetic Jaffé method. The coefficients of variation for GSSG, GSH, MDA and 8-oxo-dG were recorded at 11.4%, 4.7%, 5.5% and 11.9%, respectively.

### 2.5. Other Relevant Variables

Participants were interviewed by qualified staff to collect information on sociodemographic data, lifestyle habits and cardiovascular risk factors. Alcohol intake and smoking were classified as never, former and current status based on self-report. Physical activity was estimated in metabolic equivalents (METs) per minute/week based on standardized intensity scores [24] using reported type of activity and amount of time dedicated to each activity per week. Body mass index (BMI) was calculated using measured weight (kilograms) by height (meters) squared. Obesity was defined as a BMI equal or higher than 30 kg/m^2^. Urine cotinine was measured with an enzyme-linked immunosorbent assay (ELISA) (“Analysis DRI^®^ Cotinine” Kit, Ref. 0395 Microgenics laboratories). Concentrations below the lower limit (34 ng/mL) were detected in 77% of participants. Urine and serum creatinine were measured by the modified kinetic Jaffé method by isotope dilution mass spectrometry on a Hitachi 917 analyzer (Roche Boehringer). Urine albumin was measured by automated nephelometric immunochemistry (Behring, Germany). Renal function was assessed by the glomerular filtration rate, as estimated using the CKD-EPI equation [25].

### 2.6. Statistical Analysis

*Descriptive and association analysis.* Statistical analyses were conducted with the “survey” package of the R software (version 4.1.14) to account for the complex sampling. We reported participant characteristics and the median and interquartile range (IQR) of plasma metabolites overall and by tertiles of traffic density to compare moderate and high-to-low exposure levels. We conducted linear regression models to evaluate the association between traffic density exposure (continuous independent variable) and oxidative stress biomarkers and plasma metabolite level (dependent variables in separate models). The resultant regression coefficients were re-scaled to compare the 80th and the 20th percentiles of traffic density. All models were adjusted for sex (men, women), age (years), BMI (kg/m^2^), high education (no/yes), smoking status (never, former, current), cumulative smoking (number of pack-years), drinking status (never, former, current), glomerular filtration rate (mL/min/1.73 m^2^), physical activity (METs min/week) and urine cotinine level (mg/dL). For all the association analyses, we established a *p*-value threshold (α) of 0.05 as the statistical significance level.

*Bioinformatic exploration of potential biological implications of the findings*. For statistically significant metabolites from the association analysis, we ran Metabolite Set Enrichment Analysis (MSEA) to explore over-represented metabolites within pre-specified metabolite sets from the KEGG database [26] with MetaboAnalyst 5.0 [27]. MetaboAnalyst conducts a hypergeometric test to yield *p*-values that are interpreted as the probability of having a particular metabolite represented within a given set more than expected by chance. Subsequently, we constructed a protein interaction network. For this, we first extracted genes from the KEGG pathways with suggestively over-represented traffic-related metabolites (nominal *p*-value < 0.10). We further extended the in silico characterization of interconnections with oxidative stress by selecting additional candidate genes associated with redox balance from a published review [28]. Finally, we obtained protein–protein and protein–compound interaction networks (i.e., the interactions of proteins encoded by these genes and metabolites compounds from the IntAct database Release 243 [29] built-in feature of Cytoscape v3.9 [30]). The resulting network was filtered by removing self-loops, networks with fewer than three nodes and selecting only interactions with a Mutual Information score (MI score) of at least 0.5. Only nodes corresponding to human proteins or molecules associated with chemical functions were kept.

*Mediation analysis*. In secondary analysis, we formally tested the potential mediating role of oxidative stress as an intermediary variable in the association between traffic density with relevant metabolomic markers. Our conceptual mediation model can be found in the Supplementary File S1, Supplementary Methods. To assess natural indirect (i.e., mediated) effects, we used a counterfactual mediation framework as implemented by the multimediate R package [31]. The multimediate algorithm is able to conduct mediation analysis using the counterfactuals method. In this setting, our mediator models were separate linear models in which relevant oxidative stress biomarkers were entered as the dependent variable and traffic density (exposure) was entered as the independent variable. The outcome linear model included relevant metabolites (i.e., statistically significant in previous analysis) as the dependent variable in separate models, traffic density as the exposure and oxidative stress biomarkers as mediators. Both outcome and mediator models were adjusted for age, sex, education, BMI, smoking status, accumulated smoking (packs-year), alcohol intake, urine cotinine levels, physical activity (METs/week), triglycerides and lipid-lowering medication.

As result, absolute mediated effects (i.e., natural indirect effects) were reported as the mean difference in changes in traffic-related metabolite levels attributed to differences in oxidative stress. The direct effect was reported as the mean difference in changes in traffic-related metabolite levels not attributable to differences in oxidative stress. The total effect corresponds to the sum of the direct and the indirect effect. The relative mediated effect was calculated as the ratio between the indirect and the total effect. Confidence intervals were calculated using a resampling method based on simulations from a multivariate normal distribution [31].

## 3. Results

*Descriptive and association analysis.* Table 1 shows the crude (unadjusted) characteristics of our study population according to traffic density levels (low, moderate, high). The mean age was 52.74 years and 49.78% were women. The group exposed to the highest traffic density had higher accumulated smoking (pack-years) and was more physically active compared to less exposed subjects. Participants with higher exposure to traffic density showed lower levels of amino acids (cysteine, proline, tryptophan), products of bacterial co-metabolism (phenylpropionate) and energy metabolism (acetate) metabolite subclasses, and higher levels of cholesterol, triglycerides (LDL and IDL triglycerides), lipoprotein particle subclasses (large and medium LDL and HDL) and the oxidative stress marker GSSG/GSH% (Supplementary File S1, Supplementary Table S1). The association of traffic density (per 80th to 20th percentiles of traffic distribution comparison) with NMR metabolites (unitless) was positive for some of the measured fatty acid moieties, including CH₂CH₂CO and CH₂N (MD [95% CI] was 0.155 [0.033, 0.276] and 1.744 [0.277, 3.211], respectively). Alternatively, the association of traffic density was inverse for the amino acid cysteine (MD [95% CI] −0.010 [−0.020, −0.001]); the fatty acid isobutyrate (MD [95% CI] −0.042 [−0.077, −0.007]); some products of bacterial co-metabolism, such as trimethylamines (MD [95% CI] −0.065 [−0.113, −0.018]); acetate, a product of energy metabolism (MD [95% CI] −0.030 [−0.058, −0.002]); and the fluid-balance-associated metabolite albumin (MD [95% CI] −0.073 [−0.135, −0.011]). Regarding the association with oxidative stress biomarkers, traffic exposure was positively associated with urine GSSG/GSH (%) (3.142 [0.049, 6.236]) but inversely associated with MDA levels (nmol/mmol creatinine) (−0.097 [−0.170, −0.023]) (Table 2). All the traffic-related oxidative stress biomarkers (independent variable) were also associated with the traffic-related metabolites (dependent variables), except for the association of isobutyrate with GSSG/GSH% and that of CH₂N and acetate, and possibly cysteine, trimethylamine and albumin, with MDA (Supplementary File S1, Supplementary Table S2).

**Figure 1:**
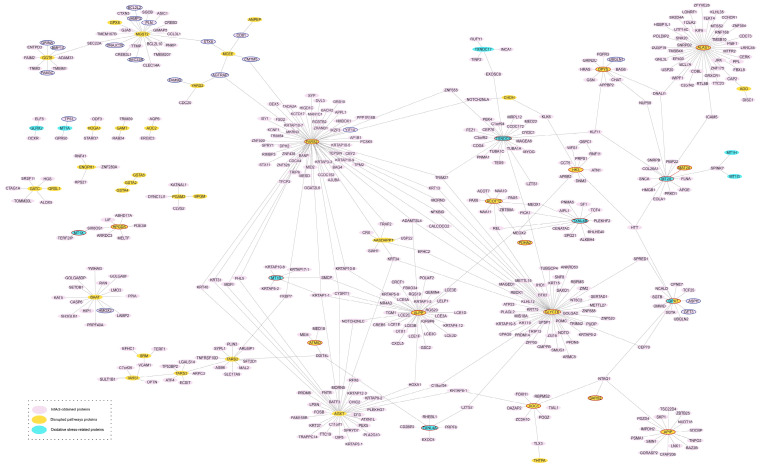
Protein interaction network generated using IntAct by redox-related proteins and pathways with over-represented traffic-exposure-associated metabolites (468 proteins connected by 493 edges). Nodes are colored according to protein data origin: oxidative-stress-related proteins are blue, proteins identified by the enrichment analysis are yellow, and IntAct-identified proteins are purple. Proteins circled in red are, at least, second-degree neighbors between the proteins of interest. Proteins circled in blue interact with a GPX protein (see Supplemental File S2, Supplemental Sheet S5).

*Bioinformatic exploration of potential biological implications of the findings*. Table 3 shows the results for the Metabolite Set Enrichment Analysis (MSEA). Valine and cysteine were the most over-represented metabolites, followed by acetate. At the *p*-value threshold of 0.10, out of the 84 KEGG-based metabolite sets included in the MSEA, 12 included over-represented statistically significant metabolites from our association analysis. These pathways were mainly associated with amino acids, carbohydrates and co-factors metabolism. The three most enriched pathways were “Pantothenate and CoA biosynthesis” (hsa00770), “Glycine, serine and threonine metabolism” (hsa00260) and “Aminoacyl-tRNA biosynthesis” (hsa00970) (Supplementary File S1, Supplementary Figure S1). Figure 1 shows the protein–protein interaction network resulting from displaying IntAct-based interactions from the list of proteins encoded by our redox-related candidate genes with proteins encoded by genes within the metabolite sets with over-represented metabolites from our association analysis (Supplementary File S2, Supplementary Sheet S1). The initial network had 1330 nodes and 4503 interactions (Supplementary File S2, Supplementary Sheet S2). We excluded 143 nodes not corresponding to human proteins or molecules associated with chemical molecules, 628 nodes with an MI score below 0.5 and 17 nodes with self-loops without identifiable ID (Supplementary File S2, Supplementary Sheet S3). Finally, the resulting protein network after filtering (Figure 1) retained a total of 468 unique proteins and 493 interactions (Supplementary File S2, Supplementary Sheet S4). Among the enriched pathways and the oxidative-stress-related proteins, 10 common proteins were found, most of which are associated with the glutathione metabolism (*GPX1* to *7*, *GSR* and *TXNDC12*), while *CAT* encodes another key enzyme involved in redox balance. Nevertheless, after filtering the interaction network, only the glutathione peroxidase *GPX7*, an endoplasmic catalase involved in the cellular response to oxidative stress, remained (Figure 1). Furthermore, only one direct interaction between a protein in a KEGG pathway with over-represented metabolites, *MAT2A* and an oxidative stress-related protein, *MT2A*, prevailed. The proteins *FARS2*, *GLYCTK*, *ALPP* and *ALAS1* are some of the largest nodes as they have the greatest number of interactions.

*Contribution of oxidative stress to traffic-density-related NMR-metabolites*. MDA accounted for 21.88 (0.19, 59.24) and 20.48 (2.26, 83.97) % of the traffic-related isobutyrate and CH_2_CH_2_CO fatty acid moiety variation, respectively. In absolute terms, of the 0.139 (0.018, 0.256) CH_2_CH_2_CO fatty acid moiety units and −0.038 (−0.0727, −0.005) isobutyrate units, according to an 80th versus the 20th percentile comparison in traffic density, 0.014 (0.003, 0.032) and −0.006 (−0.012, −0.002) were attributable to variations in MDA, respectively (Supplementary File S1, Supplementary Table S3).

## 4. Discussion

In our cross-sectional study, traffic density was positively associated with fatty acid moieties (CH_2_N, CH_2_CH_2_CO) and with markers of oxidative stress, such as GSSG/GSH in urine. On the other hand, traffic exposure was inversely associated with the amino acid cysteine and fatty acids such as isobutyrate, with products of bacterial co-metabolism (trimethylamines), energy metabolism (acetate), fluid balance metabolites (albumin) and the oxidative stress biomarker malondialdehyde (MDA). The association of traffic density with cysteine, acetate and albumin was partly explained by oxidative stress biomarkers, supporting the idea that oxidative stress is a biological intermediary in traffic-related disease. The statistical models accounted for known oxidative stress determinants, such as alcohol consumption and smoking status.

**Traffic density and metabolomics**. Evidence has shown over the years that traffic-associated air pollution (TRAP) is a known risk factor for the development of health conditions such as cardiovascular disease [32], metabolic disorders [33] and respiratory diseases, including lung cancer and asthma [34,35]. A study conducted by the University of Bari showed that exposure to TRAP produces neutrophilic airway inflammation [36]. However, the mechanisms by which TRAP causes adverse health effects still remain poorly understood, and little is known regarding the potential role of traffic exposure as a determinant of metabolite levels [37]. In our study, a broad panel of metabolites were used to assess potential metabolic traffic effects. Next, we reviewed the consistency of our results with those of other available epidemiological studies.

*Amino acids*. In our study, exposure to traffic density was inversely associated with amino acids levels, which are components of key enzymes in metabolic pathways involved in cell homeostasis, nutrition and the regulation of the immune system [38,39]. Dysfunctional levels of essential amino acids have been associated with several pathologies, such as cardiovascular and neurological disorders and certain types of cancer [40,41]. Consistently with our results, an intervention study conducted in healthy subjects (N = 43) found that exposure to PM2.5 was associated with a decrease in glutamate, aspartate and taurine levels [42]. A longitudinal study on healthy adults (N = 73) found that short-term air pollution exposure was associated with significant reductions in the levels of plasma alanine, threonine and glutamic acid [43]. A cross-sectional study (N = 54) observed an inverse association between histidine and outdoor PM2.5 level [44].

*Bacterial co-metabolism*. The gut microbiota play a vital role in human homeostasis [45], stimulate the immune system and contribute to metabolism [46]. Microbiome imbalance caused by exposure to air pollutants could have a role in cardiometabolic, infectious and inflammatory disease [47,48]. Some studies have pointed to a potential role of trimethylamines (TMAO), a component of bacterial co-metabolism involved in human physiological processes [49] and in the pathogenesis of numerous diseases, including kidney and cardiovascular diseases [47,50]. A study conducted in China (N = 114) found that oropharyngeal microbiota of healthy volunteers differed within regions of high, medium and low TRAP [51]. In addition, a pilot study conducted with obese adolescents (N = 43) demonstrated that TRAP exposure can alter the composition and abundance of the gut microbiota [52]. Consistently, in our study, traffic exposure was inversely associated with all products of bacterial co-metabolism evaluated (ethanol, methanol, isopropanol, trimethylamines and phenylpropionate).

*Fatty acids and lipoprotein subclasses.* The association of traffic density with fatty acids and most of the lipoprotein subclasses, except for HDL cholesterol, was positive, although the uncertainty of these associations was substantial. While there are no studies specifically reporting the association between traffic exposure and lipids, air pollution studies carried out in large cohorts (MESA-Air Study, META-Air Study) identified that pollutant components such as PM2.5 and NOx were positively associated with total and LDL cholesterol levels [53,54]. The fatty acid isobutyrate is also considered a by-product of bacterial co-metabolism [55,56,57].

**The role of oxidative stress in traffic-related metabolomics**. There is growing evidence in support of a role of exposure to environmental pollution in altering redox balance [58,59]. In our data, higher traffic density exposure was positively associated with GSSG/GSH levels and negatively associated with MDA levels. An increase in GSSG/GSH is indicative of increased oxidative stress at the cellular level [60,61,62], consistently with our hypotheses. Malondialdehyde is a product of lipid peroxidation, especially at the membrane level; it is derived from polyunsaturated fatty acids and increases in situations of oxidative stress [63]. Thus, identifying lower levels in subjects more exposed to traffic with respect to those less exposed was unexpected. In a small study from Cracow (N = 40), exposure to carbon monoxide (CO), a major pollutant from road traffic, was positively associated with MDA and GSSG [64]. Inconsistently with our data, short-term exposure to traffic-related black carbon concentrations in the air was positively associated with 8-isoprostane, a marker of oxidative stress in lipids [65], and 8-oxo-OhdG, a marker of oxidative stress in the cellular nucleus [66]. In our study, MDA substantially explained the association of traffic density with metabolites within the fatty acids and subproducts of bacterial co-metabolism groups. Studies on experimental models show that MDA levels were dependent on fatty acid unsaturation and correlated to carbonyls in fatty acids (CH_2_CH_2_CO) [67]. In addition, alteration in gut and lung microbiota has been observed in situations of TRAP exposure, with oxidative stress playing an important role in these alterations [68,69].

Overall, our results are consistent with the possibility that redox imbalance is a biological mediator of trafficking-related metabolic alterations, especially at the cell membrane level, and related to the microbiota [37,70].

**Bioinformatic exploration of traffic- and redox-related metabolic pathways**. The most enriched pathway was Pantothenate and CoA biosynthesis, which has been associated with mitochondrial function and energy metabolism (the most over-represented metabolites were cysteine and valine, which were decreased in the subjects most exposed to traffic density). An experimental study conducted in human epidermal keratinocytes showed that a pantothenate derivative reduced cell damage by stimulating the intracellular defense system against ROS [71]. Little is known, however, about the effect that exposure to traffic density may have on energy metabolism. In our study, higher traffic density was inversely related to acetate. Studies conducted in mice and rats also concluded that certain air pollutants, such as lead and other PM, function as deregulators of energy metabolism [55,56,57].

The molecular interactions (edges) in our protein network reflect the accumulated evidence linking proteins involved in oxidative stress and proteins in pathways with over-represented metabolites and provide an overview of the potential downstream biological implications of the most interesting findings. For instance, *MT2A* encodes for metallothionein proteins, which play an important antioxidant role and have been associated with the progression of various chronic diseases [72,73]. *MAT2A* encodes for an essential enzyme that synthesizes S-adenosylmethionine (SAM), a precursor of glutathione (GSH) [74]. Glutathione peroxidase isoform 7 (GPx7) is activated upon redox situations, mainly at the endoplasmic reticulum level, and participates in the oxidative folding of proteins [75,76]. It is known that the GPx7 structure contains cysteine [70], an amino acid that is associated with high traffic density in our data. FARS2, GLYCTK, ALAS1 and ALPP intervene in various metabolic pathways whose alteration has been associated with exposure to traffic and various pollutants [77,78,79]. For a deeper review of other interesting potential mechanisms identified in bioinformatic analysis, see Supplementary File S1, Supplementary Discussion. Given the connection of most relevant metabolites with the glutathione cycle, as a post hoc analysis, we descriptively report the interaction network of the *GPX* family of proteins (isoforms 1 to 7) with other proteins and metabolites also obtained from IntAct (Figure 1 and Supplementary File S2, Supplementary Sheet S5).

**Limitations and strengths**. Our study has several limitations. For instance, the interpretation of our findings requires some caution because the traffic exposure assessment in our study was based on the participants home address, and it does not take into account movements made throughout the day. To address this potential source of heterogeneity in the traffic exposure measurement, we used average annual traffic density as a measure of traffic exposure to select streets with a similar density and then created tertiles of traffic density to compare between low, moderate and high exposure levels, as previously done in other epidemiological studies [16]. Some other studies have employed other variables, such as nitrogen dioxide, benzene [80,81,82] and volatile organic compounds [79], or have assessed personal exposure to air pollution using rechargeable devices that can be carried by the subjects themselves or via satellite-derived data [83]. Further, it should be taken into account that humans are not only exposed to outdoor environmental pollution but also indoor pollutants (aromatic hydrocarbons, aldehydes and others) [84]. An additional limitation is related to the targeted metabolomic approach, which quantified a predefined set of metabolites. Consequently, some relevant metabolites may have been missed. Also, our findings must be interpreted with caution because diet and microbiota can affect metabolomic profiles [85]. Several sensitivity analyses have been performed including adjustment for total energy, fat, carbohydrate and protein intake from 24 h recall questionnaires, yielding essentially similar findings, suggesting that possible confounding by dietary factors is probably not relevant in our data. Last, the assumptions for mediation analysis include no unmeasured confounding by the relationship between the exposure, the outcome and the mediators, an assumption that is impossible to verify in observational studies. Another important limitation is the cross-sectional nature of our data.

Our study also has several strengths. To our knowledge, this is the first study to analyze the association between traffic density exposure and metabolomic determinations while exploring the potential role of oxidative stress as an intermediary in this association. In addition, our approach did not provide individual compound measurements but summarized the total exposure to traffic pollution, which is preferable for the purpose of our study, which included a large number of metabolites with unknown relation to traffic exposure as a whole. The strengths of this study also include the complex survey design and sample size, which allows our results to be representative for the general population of a region in Spain. Furthermore, the unique availability of a considerable panel of metabolites measured with high-quality procedures is an additional strength of the study.

## 5. Conclusions

In our study, we observed a clear association of exposure to traffic density with differences in certain metabolic patterns that have traditionally been linked to the development of chronic conditions in the general population. Our results supported the idea that oxidative stress might be a relevant mechanism of traffic-related health effects, especially for lipidic membranes and bacterial co-metabolism. Our findings need to be confirmed by prospective studies with longitudinal metabolite measurements but suggest that reinforcing public health interventions to reduce exposure to traffic in the population is needed.

## Figures and Tables

**Table 1:** Age- and gender-adjusted baseline characteristics based on traffic density (N = 1181). Mean, overall and by tertiles of traffic density.

		Traffic Density at Home Address (Cars per Day)		
	Overall	Low Traffic Density	Moderate Traffic Density	High Traffic Density
Age, years; mean	52.74	51.90	51.66	54.59
Women; %	49.78	49.21	46.23	53.72
BMI, kg/m^2^; mean	26.36	26.67	26.06	26.36
Smoking status				
Never; %	47.23	47.40	44.15	49.75
Former; %	29.53	28.39	32.71	28.50
Current; %	23.22	24.19	22.34	21.75
Cumulative smoking, pack-year; mean	9.08	8.05	9.59	9.59
Urine cotinine, mg/dL				
<12 mg/dL	77.3	95.52	77.95	78.40
12–500 mg/dL	4.73	6.05	5.1	3.08
>500 mg/dL	17.97	18.42	16.93	18.51
Alcohol intake status				
Never; %	39.26	44.73	32.79	40.10
Former; %	8.41	8.42	8.06	8.74
Current; %	52.32	46.84	56.55	51.15
eGFR, mL/min/1.73 m^2^	90.84	91.7	92.13	88.77
High education; %	72.92	69.21	78.19	72.00
Physical activity, METs min/week; mean	3135.94	3110.10	3050.08	3242.95

**Table 2:** Mean difference (95% CI) * of NMR-metabolites and oxidative stress markers when comparing the 80th and 20th percentiles of traffic density distribution in the Hortega Study (N = 1181).

Group	Metabolite	MD (95% CI)	*p*-Value
Lipoprotein profile	Cholesterol, mg/dL	1.430 (−2.340, 5.201)	0.457
	VLDL cholesterol, mg/dL	0.638 (−0.130, 1.407)	0.104
	LDL cholesterol, mg/dL	1.833 (−2.232, 5.898)	0.377
	HDL cholesterol, mg/dL	−0.832 (−2.664, 1.001)	0.374
	IDL cholesterol, mg/dL	0.487 (−0.067, 1.042)	0.085
	Total VLDL, nmol/L	0.796 (−1.492, 3.083)	0.496
	Large VLDL, nmol/L	−0.015 (−0.084, 0.054)	0.496
	Medium VLDL, nmol/L	0.002 (−0.459, 0.463)	0.994
	Small VLDL, nmol/L	0.808 (−1.252, 2.869)	0.442
	Total LDL, nmol/L	17.08 (−22.022, 56.182)	0.392
	Large LDL, nmol/L	2.497 (−3.041, 8.035)	0.377
	Medium LDL, nmol/L	11.853 (−6.439, 30.146)	0.204
	Small LDL, nmol/L	2.830 (−17.158, 22.819)	0.781
	Total HDL, nmol/L	−0.311 (−1.207, 0.524)	0.440
	Large HDL, µmol/L	0.002 (−0.006, 0.010)	0.695
	Medium HDL, µmol/L	0.004 (−0.301, 0.309)	0.979
	Small HDL, µmol/L	−0.347 (−0.966, 0.271)	0.271
Amino acids	Alanine	0.013 (−0.058, 0.084)	0.718
	Creatine phosphate	−0.008 (−0.021, 0.004)	0.187
	Creatine	−0.010 (−0.021, 0.002)	0.090
	Cysteine	−0.010 (−0.020, −0.001)	0.038
	Glutamine	−0.008 (−0.063, 0.047)	0.772
	Proline	0.017 (−0.044, 0.078)	0.595
	Tryptophan	0.017 (−0.026, 0.060)	0.432
	Tyrosine	−0.014 (−0.036, 0.007)	0.198
	Isoleucine	0.004 (−0.042, 0.049)	0.875
	Leucine	−0.002 (−0.040, 0.036)	0.909
	Valine	−0.038 (−0.083, 0.006)	0.093
Inflamation marker	N-acetylglutamine	−0.031 (−0.068, 0.007)	0.109
Fatty acids	CH_2_CH_2_CO	0.155 (0.033, 0.276)	0.013
	CH_2_CH_3_	0.121 (−0.033, 0.274)	0.123
	CH_2_N	1.744 (0.277, 3.211)	0.020
	CH_3_	0.063 (−0.358, 0.483)	0.770
	CHCH_2_CH	0.005 (−0.095, 0.105)	0.919
	Isobutyrate	−0.042 (−0.077, −0.007)	0.020
Products of bacterial co-metabolism	Ethanol	−0.004 (−0.196, 0.187)	0.964
	Isopropanol	−0.041 (−0.087, 0.006)	0.087
	Methanol	−0.012 (−0.024, 0.001)	0.065
	Trimethylamines	−0.065 (−0.113, −0.018)	0.008
	Phenylpropionate	0.030 (−0.039, 0.098)	0.395
	O-phosphoethanolamine	−0.036 (−0.079, 0.008)	0.111
Energy metabolism	*Glycolisis*		
	Citrate	−0.024 (−0.060, 0.013)	0.208
	Lactate	0.299 (−0.065, 0.663)	0.108
	Pyruvate	0.001 (−0.013, 0.014)	0.905
	*Ketone bodies*		
	Acetate	−0.030 (−0.058, −0.002)	0.034
	Acetone	0.062 (−0.002, 0.127)	0.057
	3-Hydroxybutyrate	−0.006 (−0.063, 0.050)	0.822
Fluid balance	Albumin	−0.073 (−0.135, −0.011)	0.022
	Creatinine	−0.006 (−0.027, 0.015)	0.572
Oxidative stress markers	GSSG/GSH,%	3.142 (0.049, 6.236)	0.047
	Malondialdehyde (MDA), nmol/mmol creatinine	−0.097 (−0.170, −0.023)	0.010
	8-oxo-7,8-dihydroguanine (8-oxo-dG), nmol/mmol creatinine	0.116 (−0.068, 0.300)	0.216

Models were adjusted for age, sex, education, BMI, smoking status, cigarette packages per year, alcohol intake, urine cotinine levels, physical activity per week, triglycerides and lipid-lowering medication. * We normalized the spectral vector to the total spectral area, excluding residual water signals to minimize the effects of variable dilution of the sample. The metabolic content is therefore expressed in relative metabolic content (unitless), unless other units are indicated.

**Table 3:** Metabolite Enrichment Analysis results for the significant metabolites associated with traffic density.

KEGG-Based Pathways	Total	Expected	Hits	Enrich.	Raw	Holm	FDR	Metabolites
				Ratio	*p*-Value	*p*-Value	*p*-Value	
Pantothenate and CoA biosynthesis	19	0.050	2	40.404	0.001	0.072	0.072	Cysteine, Valine
Glycine, serine and threonine metabolism	33	0.086	2	23.283	0.003	0.217	0.110	Creatine, Cysteine
Aminoacyl-tRNA biosynthesis	48	0.125	2	16.000	0.006	0.452	0.154	Cysteine, Valine
Thiamine metabolism	7	0.018	1	54.945	0.018	1	0.290	Cysteine
Valine, leucine and isoleucine biosynthesis	8	0.021	1	48.077	0.021	1	0.290	Valine
Taurine and hypotaurine metabolism	8	0.021	1	48.077	0.021	1	0.290	Cysteine
Pyruvate metabolism	22	0.057	1	17.452	0.056	1	0.636	Acetate
Glycolysis/gluconeogenesis	26	0.068	1	14.771	0.066	1	0.636	Acetate
Glutathione metabolism	28	0.073	1	13.717	0.071	1	0.636	Cysteine
Glyoxylate and dicarboxylate metabolism	32	0.083	1	12.005	0.081	1	0.636	Acetate
Cysteine and methionine metabolism	33	0.086	1	11.641	0.083	1	0.636	Cysteine
Arginine and proline metabolism	38	0.099	1	10.101	0.095	1	0.648	Creatine

Total is the total number of compounds in the pathway; Expected is the number of matched compounds expected by chance given the pathway size; Hits is the number of matched compounds from the data; Enrichment Ratio is the number of hits divided by the expected number of hits. Raw *p*-value calculated from the enrichment analysis; Holm *p*-value was adjusted using the Holm–Bonferroni method; FDR *p*-value was adjusted using False Discovery Rate (FDR); metabolites showed over-represented statistically significant metabolites from the association analysis.

## Data Availability

The data presented in this study are available on request to the corresponding author, upon a reasonable request. The data are not publicly available because unrestricted data sharing is not possible due to privacy or ethical restrictions.

## Supplementary Materials

The following supporting information can be downloaded at: https://www.mdpi.com/article/10.3390/antiox12122122/s1. References [86,87,88,89,90,91,92,93,94,95,96,97,98,99,100,101,102,103,104,105,106,107,108,109,110,111,112,113,114,115,116,117,118,119,120,121,122] are cited in the Supplementary Materials.
