# Supplementary material for: Traffic Density Exposure, Oxidative Stress Biomarkers and Plasma Metabolomics in a Population-Based Sample: The Hortega Study
Source: Antioxidants (Basel). 2023 Dec 15;12(12):2122. doi: 10.3390/antiox12122122 (PMC10740723; doi:10.3390/antiox12122122)
Supplement: Supplementary file 1 [file antioxidants-12-02122-s001.zip › antioxidants-2751613-supplementary File S1.pdf]

SUPPLEMENTARY MATERIAL

Traffic density exposure, oxidative stress biomarkers and plasma metabolomics in a population-based sample: The Hortega Study

Laura Sanchez-Rodriguez, Marta Galvez-Fernandez, Ayelén Rojas-Benedicto, Arce Domingo-Relloso, Nuria Amigo, Josep Redon, Daniel Monleon, Guillermo Saez, Maria Tellez-Plaza, Juan Carlos Martin-Escudero, Rebeca Ramis

Table of contents

Supplementary Methods **2**
Supplementary Tables **4**
Supplementary Table S1. 4
Supplementary Table S2. 7
Supplementary Table S3. 8
Supplementary Figures **8**
Supplementary Discussion **10**
Supplementary Findings 1 10
Supplementary Findings 2 12
Supplementary References **15**

Supplementary Methods

Conceptual framework for mediation analysis

The aim of causal mediation analysis is to find out how much of the total effect following a well-defined exposure is partly due to an intermediate variable involved in the causal pathway (indirect effect). In our mediation framework, the exposure of interest is traffic density (TD), which has been shown to be associated with several health outcomes, and the intermediate variables we are specifically interested in investigating are the oxidative stress (OS) biomarkers GSSG/GSH and MDA.

In other words, the question is whether the effect of an intervention to change traffic exposure on metabolite levels can be explained (i. e. mediated) by oxidative stress, after adjusting for confounders. Our conceptual model can easily be presented in the attached DAG.

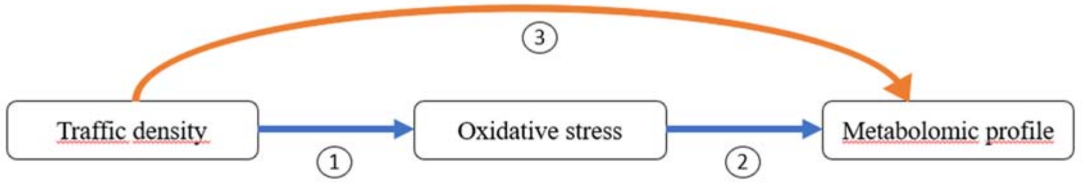

In this DAG, exposure to traffic can cause a situation of oxidative stress through the alteration of the redox signal (arrow 1). The alteration of metabolite levels due to oxidative stress is well supported based on the epidemiological literature supporting oxidative stress biomarkers as being responsible for the alteration of various metabolic pathways (arrow 2). In addition, exposure to traffic and other environmental pollutants has also been directly associated with changes in metabolomic profile (arrow 3). However

we do not have experimental, but, observational, data. Thus, caution must be made by the readers when interpreting our mediation results, which are a secondary analysis in our study.

Supplementary Tables

Supplementary Table S1. Median (IQR) NMR-metabolites and oxidative stress markers levels by traffic density categories in Hortega Study participants (n = 1181)

			Traffic density in home address		
**Group**	**Metabolite**	**Overall**	**Low Traffic density**	**Moderate Traffic density**	**High Traffic density**
**n (%)**		1181 (100)	405 (34)	376 (32)	400 (34)
**Lipoprotein profile**	*Cholesterol, mg/dL*	196 (173 - 223)	192 (173 - 223)	196 (173 - 227)	200 (177 - 223)
	VLDL cholesterol	17.90 (8.58 - 28.81)	16.60 (7.85 - 26.10)	18.43 (9.19 - 30.02)	17.57 (8.95 - 30.52)
	LDL cholesterol	139.34 (116.18 - 167.31)	139.61 (116.60 - 164.01)	139.10 (114.10 - 166.52)	139.64 (117.60 - 168.67)
	HDL cholesterol	68.60 (55.85 - 81.71)	68.11 (55.38 - 80.04)	69.28 (56.17 - 83.15)	68.83 (55.59 - 82.62)
	IDL cholesterol	10.65 (6.72 - 15.412)	9.74 (6.321 - 13.72)	10.88 (7.03 - 15.64)	11.23 (7.09 - 16.089)
	Triglycerides, mg/dL	150 (106 - 212)	141 (97 - 203)	159 (106 - 221)	150 (106 - 212)
	VLDL triglycerides	81.75 (45.01 - 140.24)	75.80 (43.65 - 131.06)	90.53 (46.30 - 154.38)	79.30 (45.57 - 136.70)
	LDL triglycerides	23.37 (18.80 - 29.16)	22.68 (18.24 - 28.01)	23.54 (18.85 - 29.73)	23.91 (19.49 - 29.77)
	HDL triglycerides	14.25 (10.83 - 18.63)	13.70 (10.54 - 17.37)	15.01 (10.92 - 19.17)	14.55 (11.27 - 18.66)
	IDL triglycerides	12.98 (10.02 - 16.73)	12.18 (9.86 - 16.00)	13.18 (10.17 - 16.98)	13.47 (10.08 - 17.22)
	Lipoprotein particle concentration				
	Large VLDL. nmol/L	1.25 (0.72 - 2.28)	1.17 (0.71 - 2.11)	1.43 (0.76 - 2.51)	1.23 (0.68 - 2.24)
	Medium VLDL. nmol/L	6.82 (3.97 - 11.62)	6.50 (3.72 - 11.25)	7.52 (4.21 - 12.51)	6.76 (4.09 - 11.20)
	Small VLDL. nmol/L	52.12 (27.65 - 89.71)	47.61 (26.90 - 82.43)	58.56 (28.85 - 98.67)	50.96 (27.04 - 90.30)

	Large LDL. nmol/L	217.25 (182.62 - 252.95)	215.66 (184.22 - 250.62)	218.16 (180.56 - 251.61)	219.10 (183.85 - 255.45)
	Medium LDL. nmol/L	499.08 (404.95 - 623.47)	496.12 (409.79 - 620.42)	493.85 (393.35 - 634.64)	505.35 (408.15 - 627.87)
	Small LDL. nmol/L	687.92 (563.78 - 857.27)	686.14 (564.87 - 837.73)	707.82 (565.76 - 873.34)	675.15 (560.68 - 853.05)
	Large HDL. µmol/L	0.33 (0.28 - 0.38)	0.32 (0.28 - 0.37)	0.33 (0.28 - 0.39)	0.34 (0.29 - 0.39)
	Medium HDL. µmol/L	10.77 (8.80 - 13.03)	10.43 (8.78 - 12.61)	10.88 (8.65 - 13.24)	10.95 (8.88 - 13.07)
	Small HDL. µmol/L	23.50 (19.78 - 27.62)	22.92 (19.70 - 27.28)	24.23 (20.58 - 27.84)	23.62 (9.31 - 27.54)
**Amino acids**	Alanine	16.48 (15.75 - 17.03)	16.53 (15.80 - 17.01)	16.44 (15.65 - 17.10)	16.45 (15.79 - 16.98)
	Creatine phosphate	1.65 (1.54 - 1.73)	1.66 (1.57 - 1.733)	1.64 (1.53 - 1.73)	1.65 (1.53 - 1.73)
	Creatine	1.86 (1.74 - 1.96)	1.87 (1.77 - 1.97)	1.85 (1.72 - 1.96)	1.85 (1.73 - 1.94)
	Cysteine	1.40 (1.31 - 1.48)	1.42 (1.33 - 1.50)	1.39 (1.30 - 1.47)	1.38 (1.30 - 1.47)
	Glutamine	7.57 (7.10 - 8.00)	7.59 (7.18 - 8.01)	7.56 (7.06 - 8.04)	7.56 (7.04 - 7.94)
	Proline	9.44 (8.90- 9.90)	9.44 (8.96 - 9.87)	9.44 (8.90 - 9.98)	9.40 (8.87 - 9.861)
	Tryptophan	2.13 (1.94 - 2.34)	2.14 (1.93 - 2.33)	2.15 (1.942 - 2.37)	2.11 (1.95 - 2.31)
	Tyrosine	2.66 (2.48 - 2.80)	2.67 (2.52 - 2.82)	2.65 (2.47 - 2.80)	2.66 (2.48 - 2.79)
	Isoleucine	11.57 (11.16 - 11.86)	11.61 (11.20 - 11.90)	11.55 (11.13 - 11.83)	11.55 (11.15 - 11.83)
	Leucine	10.09 (9.71 - 10.41)	10.12 (9.75 - 10.43)	10.06 (9.67 - 10.39)	10.10 (9.68 - 10.38)
	Valine	10.95 (10.49 - 11.27)	11.03 (10.57 - 11.32)	10.93 (10.47 - 11.26)	10.91 (10.44 - 11.27)
**Inflamation marker**	N-acetylglutamine	9.17 (8.77 - 9.46)	9.20 (8.87 - 9.48)	9.12 (8.7 - 9.47)	9.18 (8.72 - 9.45)
**Fatty acids**	CH_2CH_2CO	18.53 (17.65 - 19.99)	18.33 (17.53 - 19.67)	18.80 (17.72 - 20.27)	18.69 (17.68 - 19.98)
	CH_2CH_3	37.90 (36.91 - 39.142)	37.73 (36.69 - 38.83)	38.06 (37.01 - 39.36)	37.92 (36.98 - 39.31)
	CH_2N	142.45 (129.61 - 159.98)	138.48 (128.93 - 155.55)	144.64 (130.16 - 162.95)	144.43 (130.86 - 162.06)
	CH_3	80.78 (78.46 - 83.04)	81.01 (78.65 - 82.93)	80.73 (78.17 - 83.12)	80.66 (78.53 - 83.10)

	$CHCH_2CH$	12.45 (11.92 - 13.09)	12.52 (11.91 - 13.09)	12.35 (11.86 - 13.05)	12.54 (11.96 - 13.15)
	Ethanol	9.11 (8.50 - 9.73)	9.06 (8.51 - 9.79)	9.15 (8.50 - 9.72)	9.10 (8.53 - 9.72)
	Isobutyrate	7.04 (6.68 - 7.30)	7.09 (6.76 - 7.33)	7.00 (6.65 - 7.27)	7.00 (6.62 - 7.29)
**Products of bacterial co-metabolism**	Isopropanol	6.44 (6.07 - 6.78)	6.47 (6.16 - 6.75)	6.4 (6.0 - 6.81)	6.42 (6.03 - 6.78)
	Methanol	0.73 (0.67 - 0.80)	0.74 (0.69 - 0.81)	0.73 (0.67 - 0.80)	0.73 (0.67 - 0.79)
	Trimethylamines	6.47 (6.02 - 6.86)	6.57 (6.17 - 6.95)	6.43 (5.94 - 6.86)	6.43 (6.00 - 6.80)
	Phenylpropionate	13.34 (12.84 - 13.84)	13.34 (12.83 - 13.80)	13.39 (12.79 - 13.89)	13.29 (12.88 - 13.82)
	O-phosphoethanolamine	6.06 (5.69 - 6.38)	6.11 (5.80 - 6.38)	6.02 (5.63 - 6.35)	6.02 (5.67 - 6.38)
**Energy metabolism**	*Glycolisis*				
	Citrate	4.49 (4.17 - 4.75)	4.54 (4.24 - 4.76)	4.44 (4.13 - 4.76)	4.47 (4.12 - 4.74)
	Lactate	20.13 (17.99 - 23.23)	19.56 (17.72 - 22.50)	20.43 (18.25 - 23.65)	20.48 (18.10 - 23.46)
	Pyruvate	1.78 (1.68 - 1.88)	1.78 (1.70 - 1.88)	1.78 (1.67 - 1.89)	1.78 (1.67 - 1.88)
	Ketone bodies				
	Acetate	5.57 (5.30 - 5.78)	5.59 (5.38 - 5.78)	5.56 (5.23 - 5.81)	5.55 (5.24 - 5.75)
	Acetone	6.44 (5.88 - 7.29)	6.33 (5.81 - 7.24)	6.55 (5.95 - 7.34)	6.44 (5.89 - 7.28)
	3-Hydroxybutyrate	7.92 (7.41 - 8.35)	7.93 (7.47 - 8.32)	7.89 (7.38 - 8.37)	7.89 (7.34 - 8.35)
**Fluid balance**	Albumin	9.55 (8.97 - 10.09)	9.67 (9.13 - 10.15)	9.48 (8.83 - 10.10)	9.51 (8.89 - 9.99)
	Creatinine	3.12 (2.98 - 3.22)	3.12 (2.99 - 3.23)	3.11 (2.97 - 3.22)	3.11 (2.97 - 3.21)
**Oxidative stress markers**	Glutathione ratio (GSSG/GSH), %	4.22 (2.62 - 6.27)	4.14 (2.50 - 6.43)	4.22 (2.56 - 6.40)	4.32 (2.86 - 5.98)
	Malondialdehyde (MDA), nmol/mmol creatinine	0.58 (0.34 - 0.82)	0.62 (0.35 - 0.84)	0.53 (0.31 - 0.82)	0.59 (0.35 - 0.81)
	8-oxo-7.8-dihydroguanine (8-oxo-dG), nmol/mmol creatinine	2.66 (1.96 - 3.66)	2.55 (1.93 - 3.56)	2.66 (1.98 - 3.66)	2.66 (1.97 - 3.66)

Metabolites previously corrected by fasting time (hours). Except for the lipoprotein subclasses, metabolites were unitless because normalized the spectral vector to the total spectral vector to the total spectral area excluding residual water signals to minimize the effects of variable dilution of the sample. The metabolic content is therefore expressed as relative metabolic content.

Supplementary Table S2. Mean difference (95% CI) of NMR-metabolites comparing the 20th to the 80th percentiles of traffic density-associated oxidative stress biomarkers.

		Glutathione ratio (GSSG/GSH)		Malondialdehyde (MDA)	
**Group**	**Metabolite**	**MD (95% CI)**	**p-value**	**MD (95% CI)**	**p-value**
Amino acids	Cysteine	0.001 (0.000, 0.001)	0.017	0.005 (0.000, 0.011)	0.070
Fatty acids	CH_2CH_2CO	-0.009 (-0.016, -0.002)	0.013	-0.102 (-0.157, -0.047)	0.000
	CH_2N	-0.099 (-0.176, -0.023)	0.011	0.070 (-0.556, 0.697)	0.826
	Isobutyrate	0.001 (-0.002, 0.003)	0.675	0.044 (0.019, 0.069)	0.001
Products of bacterial co-metabolism	Trimethylamines	0.006 (0.004, 0.009)	0.000	0.003(-0.003, 0.048)	0.089
Energy metabolism	Acetate	-0.002 (-0.003, 0.000)	0.035	0.005 (-0.005, 0.016)	0.301
Fluid balance	Albumin	0.006 (0.003, 0.009)	0.000	0.023 (-0.003, 0.048)	0.089

The dependent variable were the traffic density-related metabolites. The mean difference is the linear regression coefficient for traffic density related oxidative stress biomarkers from Table 2. Model was adjusted for age, sex, education, BMI, smoking status, cigarette packages per year, alcohol intake, urine cotinine levels, physical activity per week, triglycerides and lipid-lowering medication.

Supplementary Table S3. Differences in traffic density-related NMR-metabolites attributable to differences in oxidative stress biomarkers ('mediated effects') in the Hortega Study (N=1181).

		GSSG/GSH		MDA	
**Plasma Metabolite**	**Total Effect**	**Mediated effect**	**Percent explained, %**	**Mediated effect**	**Percent explained, %**
Cysteine	-0.010 (-0.019, -0.001)	0.000 (-0.000, 0.001)	-3,76 (-28.60, 3.98)	-0.001 (-0.020, 0.000)	10.90 (-2.14, 60.22)
CH2CH2CO	0.139 (0.018, 0.256)	-0.007 (-0.019, 0.001)	-2.40 (-23.41, 0.88)	0.014 (0.003, 0.032)	21.88 (0.19, 59.24)
CH2N	1.736 (0.412, 3.123)	-0.060 (-0.182, 0.026)	-4.43 (-19.96, 1.99)	-0.019 (-0.154, 0.104)	-1.50 (-15.05, 8.52)
Isobutyrate	-0.038 (-0.0727, -0.005)	0.001 (-0.001, 0.003)	-3.14 (-17.34, 6.06)	-0.006 (-0.012, -0.002)	20.48 (2.26, 83.97)
Trimethylamines	-0.060 (-0.107, -0.017)	0.004 (-0.000, 0.010)	-8.68 (-31.74, 0.42)	-0.003 (-0.008, 0.001)	5.92 (-1.78, 19.89)
Acetate	-0.020 (-0.050, 0.010)	-0.001 (-0.004, 0.000)	9.75 (-40.90, 74.21)	0.001 (-0.004, 0.002)	1.45 (-74.40, 49.34)
Albumin	-0.066 (-0.127, -0.010)	0.0032 (-0.001, 0.010)	5.56 (-26.65, 2.98)	-0.002 (-0.008, 0.004)	4.06 (-8.35, 27.54)

Mediated effects are calculated based on the counterfactual framework, i.e. leaving the exposure constant and substracting the NMR-metabolites units with oxidative stress biomarkers fixed to the value they would take when exposed to the 80th percentile of traffic density to the NMR-metabolites units with oxidative stress biomarkers fixed to the value they would take when exposed to the 20th percentile of traffic density [Y (E, M(e)) – Y [E, M(e*)), being e the 80th percentile of traffic density and e* the 20th percentile]. Mediated percentages are calculated as the rate between the mediated effect and the total effect. Model adapted from Jerolon et al. 2021. Selected metabolites were adjusted for age, sex, education, BMI, smoking status, cigarette packages per year, alcohol intake, urine cotinine levels, physical activity per week, triglycerides and lipid-lowering medication. We normalized the spectral vector to the total spectral area excluding residual water signals to minimize the effects of variable dilution of the sample. The metabolic content is therefore expressed in relative metabolic content (unitless).

Supplementary Figures

Supplementary Figure S1. Network view for the Enriched Metabolite Sets adapted from MetaboAnalyst 5.0. Each node represents a metabolite set with its colour based on its Raw p-value, and its size is based on fold enrichment (hits/expected metabolites). Two metabolite sets are connected by an edge if the number of their shared metabolites is over 25% of the total number of their combined metabolite.

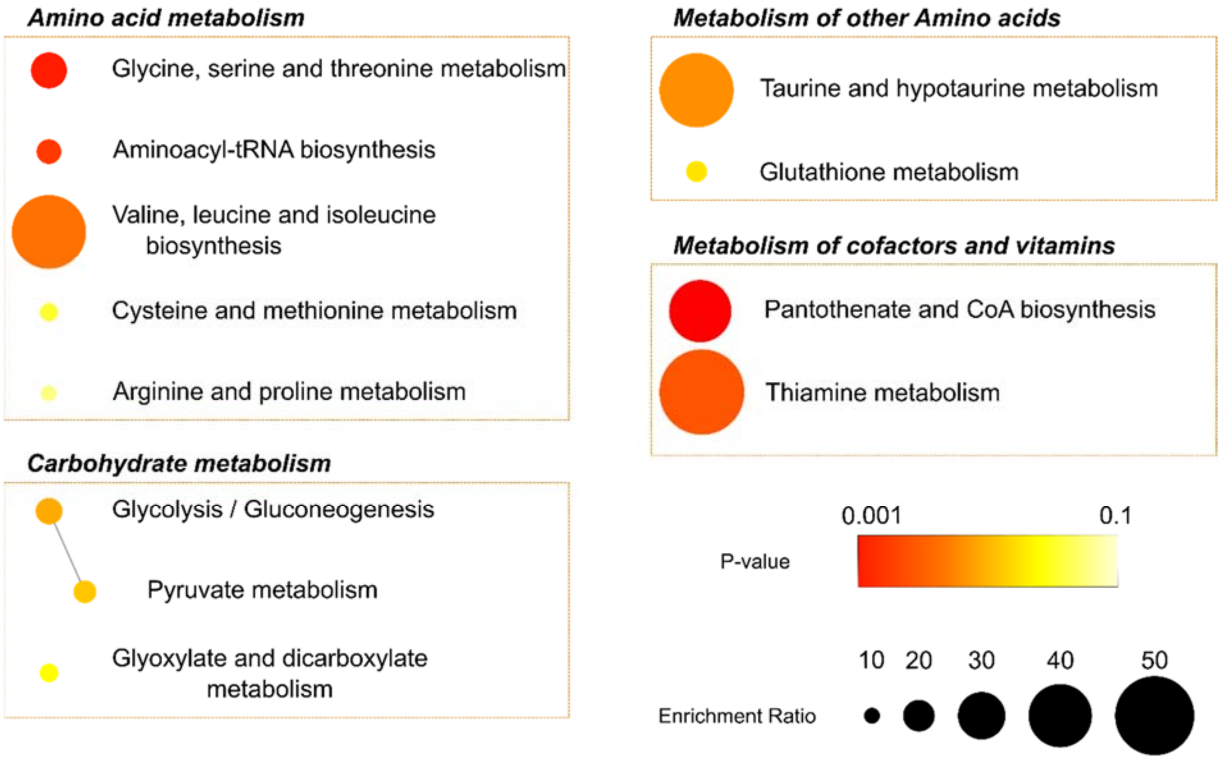

Supplementary Discussion

Supplementary Findings 1: Bioinformatic exploration of traffic and redox-related metabolic pathways.

Several pathways related to amino acids, carbohydrates and co-factors metabolism were identified in enrichment analysis (Table 3). The most enriched pathway was Pantothenate and CoA biosynthesis. Pantothenate and CoA levels have been linked to mitochondrial function and energy metabolism. In cell physiology, pantothenic acid (B5) is a precursor in the synthesis of coenzyme A and is also essential for alpha-ketoglutarate and pyruvate dehydrogenase complexes, as well as for mitochondrial energy metabolism [1]. Coenzyme A (CoA) is an essential cofactor that plays a central role in numerous metabolic pathways such as fatty acid oxidation, ketogenesis, fatty acid biosynthesis, amino acid metabolism, among others [2]. Studies have shown that alteration of this metabolic pathway is associated with the onset and development of degenerative disorders, as well as cellular necrosis and apoptosis processes induced by cholesterol oxidation [3,4].

Interestingly, in our enrichment analysis, the most over-represented metabolites cysteine and valine were associated to the pathway of pantothenate and CoA biosynthesis, both of which appear decreased in the subjects most exposed to traffic density. experimental study conducted in 2018 with human epidermal keratinocytes studied the effects of a pantothenic acid derivative on an environmental pollutant that generates reactive oxygen species (ROS), responsible for oxidative stress. The study showed that the derivative reduced cell damage by stimulating the intracellular defence system against ROS [5].

The second most enriched pathway was the glycine, serine and threonine metabolism, which is involved in multiple biochemical processes, including protein synthesis or energy production [6,7]. In our enrichment analysis, cysteine and creatine were the most represented metabolites associated to this pathway and whose levels were decreased in the most traffic-exposed subjects. Literature links exposure to heavy metals or oxidative stress with an overexpression of Akt kinase, an important regulator of fuel metabolism

that requires glycine and serine for its proper function [8]. Therefore, it is to be expected that in a situation that generates oxidative stress the amino acid levels involved in this metabolic pathway and in the synthesis of Akt kinase are decreased, as is the case in our analysis of creatine and cysteine.

Another enriched pathway was aminoacyl-tRNA biosynthesis, which binds amino acids to transfer RNA (tRNA) and thus contributes to protein synthesis and peptide chain elongation [9]. This pathway was associated to the levels of cysteine and valine, lower plasma levels of this metabolites were associated to higher traffic density exposure. There is evidence that links the disruption of this metabolic pathway to the onset and development of cardiac and metabolic diseases as well as the progression of certain tumours such as breast cancer [10,11]. Although there is scarce literature that has evaluated the relationship between the alteration of this pathway to the exposure to traffic density, a cross-sectional study carried out in 2008 showed a strong epidemiological association between exposure to environmental pollutants and the development of insulin-resistant diabetes through the alteration of protein synthesis and the regulation of lipid metabolism [12]. An experimental study conducted in Japan with rat cells found that exposure to certain environmental pollutants disrupted tRNA biosynthesis [13].

Other mitochondrial energy-related pathways, such as Pyruvate metabolism and Glycolysis/Gluconeogenesis, were identified in the enrichment analysis. Both pathways are closely related, as they are opposing processes that regulate glucose levels in the body. Pyruvate plays an essential role in central carbon metabolism. It is generated from various sources, such as lactate oxidation, alanine transamination or as a terminal product of glycolysis. Disruption of its metabolism has been shown to play a role in the development of diseases such as cancer, neurodegenerative disorders and cardiac pathologies [14].

Another enriched pathway was Glyoxylate and dicarboxylate metabolism, involved in carbohydrate biosynthesis from fatty acids or CoA-containing precursors [15]. The metabolite acetate was the most represented metabolite linked to this pathway, being inversely associated with traffic density exposure. Studies have shown the effects of exposure to environmental pollutants on these metabolites levels, but no

clear association has been established. One study in mice looked at the effect of PM2.5 exposure on their metabolome and found that the most exposed mice had alterations in energy metabolism, including dysregulation of the glyoxylate and dicarboxylate pathway [16].

Supplementary Findings 2: Biological implications of most connected nodes.

Among the nodes with most interactions and highest MI score, which represents the degree of confidence for the interactions, potentially interesting information can be retrieved.

The *FARS2* gene encodes for the enzyme aminoacyl-tRNA synthetase and its deficiency has been associated with numerous mitochondrial diseases [17]. Alterations in its expression have been associated with oxidative stress [1]. This protein was found to have the highest number of interactions in the network. Within this network, the interactions with the highest MI score were to APPL1 and CALCOCO2 proteins. APPL1 plays a central role as a contributor to adiponectin and insulin signaling, its expression is altered in response to numerous stimuli [18]. Although the effect of traffic density on its expression has not been studied, it is known that exposure to PM2.5 increases insulin resistance and promotes metabolic dysfunction, so that APPL1 expression might be expected to be altered in these situations [19]. In addition, our Metabolite Enrichment Analysis shows that the central pathway of carbohydrate metabolism is altered at high traffic density exposures. Whereas CALCOCO2 is an autophagy receptor that causes mitochondrial damage under oxidative stress by promoting mitophagy [20,21].

The second protein that shows the most interactions in the resulting network is GLYCTK, which codes for a kinase involved in numerous metabolic pathways such as Pentose phosphate pathway; Glycine, serine and threonine metabolism or Glyoxylate and dicarboxylate metabolism [22], the latter two associated with traffic. A study conducted in *Arabidopsis thaliana* Columbia ecotype seeds showed that herbicide exposure led to oxidative stress through, among others, alteration of the pentose phosphate pathway [23]. TRIP13, DTX2 and GOLGA2 shown the strongest interactions with this GLYCTK. The protein is involved in several cellular processes, including cell cycle regulation and DNA repair [24]. Environmental stressors

such as UV radiation or cellular stress have been shown to overexpress *TRIP13* [25]. DTX2 plays an important role in post-transcriptional modifications of proteins, as well as in other pathways such as the Notch signalling pathway, overexpression of which leads to oxidative stress [26]. GOLGA2 is a golgin, a protein localized in the Golgi apparatus involved in its maintenance and vesicular transport. Although is not clearly linked to oxidative stress, its being associated with autophagosome formation with is usually linked to oxidative damage and stress conditions [27].

Another MSEA protein that has shown a large number of interactions is ALAS1, which is involved in heme group synthesis and normal mitochondrial function [28]). Consistent with our results, a study in rats showed that exposure to a toxic environmental pollutant (2,3,7,8-tetrachlorodibenzo-p-dioxin) resulted in suppression of ALAS1 [29]. The strongest interaction with this node was with the DUSP19 protein, a dual-specificity phosphatase involved in the regulation of numerous biological processes, such as cell differentiation or the response to oxidative stress through activation of the MAPK signalling cascade [30]. Research conducted in 2020 on male mice showed that exposure to microplastics generates oxidative stress through activation of this pathway [31]. A highly significant interaction was also observed between ALAS1 and CDC73, a protein part of the transcriptional machinery and whose alteration has been associated with diseases of the parathyroid gland, such as primary hyperparathyroidism (PHPT) [32]. A study carried out in Poland looked at the presence or absence of oxidative stress in 52 patients with PHPT and concluded that there was an increase in reactive oxygen species [33]. These results are consistent with the obtained network and the interactions found between these three proteins ALAS, DUSP19 and CDC73.

The ALPP protein also has a large number of interactions, which has an important role in phosphorus and calcium metabolism and its expression can be altered by certain environmental pollutants such as tobacco smoke [34,35]. The strongest interaction was with KRTAP5-9, which is involved in the formation and maintenance of hair structure [36]. However, there is no scientific evidence available that has studied its association with oxidative stress or exposure to environmental pollutants. Interaction is also observed between ALPP and another protein from the same family as the previous one, KRTAP1-1. In the

network we also found that KRTAP1-1 interacts with MT1B, a protein associated by Gelain to oxidative stress. It plays important roles in protection against heavy metal toxicity, DNA damage and oxidative stress. The protein is part of the metallothionein family, which has been associated with tumour formation and progression [37]. A study of 218 lung cancer patients associated environmental exposure to chromium with alterations in MT1B expression [38], among other genes. KRTAP1-1, in addition to ALPP, also interacts with AGXT, which plays an important role in the metabolism of glyoxylate [39], one of the pathways associated with traffic in our analysis. In the network, it showed a high confidence interaction with PEX5, which is to be expected as both are known to be involved in the function of the peroxisome, involved in the metabolism of reactive oxygen species [40].

Finally, the peroxidase GPX7 is the only common protein among the enriched pathways and the oxidative-stress related proteins that remains after the network curation. GPX7 has a known role in the maintenance of redox homeostasis and experimental animal studies have shown that alteration of its expression can lead to various pathological conditions such as neurodegeneration and carcinogenesis [41,42]. No studies have evaluated its role in traffic exposure.

Supplementary References

1. Depeint, F.; Bruce, W.R.; Shangari, N.; Mehta, R.; O'Brien, P.J. Mitochondrial Function and Toxicity: Role of the B Vitamin Family on Mitochondrial Energy Metabolism. *Chem. Biol. Interact.* **2006**, *163*, 94–112, doi:10.1016/j.cbi.2006.04.014.
2. LEONARDI, R.; JACKOWSKI, S. Biosynthesis of Pantothenic Acid and Coenzyme A. *EcoSal Plus* **2007**, *2*, 10.1128/ecosalplus.3.6.3.4, doi:10.1128/ecosalplus.3.6.3.4.
3. Santambrogio, P.; Ripamonti, M.; Cozzi, A.; Raimondi, M.; Cavestro, C.; Di Meo, I.; Rubio, A.; Taverna, S.; Tiranti, V.; Levi, S. Massive Iron Accumulation in PKAN-Derived Neurons and Astrocytes: Light on the Human Pathological Phenotype. *Cell Death Dis.* **2022**, *13*, 1–12, doi:10.1038/s41419-022-04626-x.
4. Yamanaka, K.; Urano, Y.; Takabe, W.; Saito, Y.; Noguchi, N. Induction of Apoptosis and Necroptosis by 24(S)-Hydroxycholesterol Is Dependent on Activity of Acyl-CoA:Cholesterol Acyltransferase 1. *Cell Death Dis.* **2014**, *5*, e990–e990, doi:10.1038/cddis.2013.524.
5. Yokota, M.; Yahagi, S.; Masaki, H. Ethyl 2,4-Dicarboethoxy Pantothenate, a Derivative of Pantothenic Acid, Prevents Cellular Damage Initiated by Environmental Pollutants through Nrf2 Activation. *J. Dermatol. Sci.* **2018**, *92*, 162–171, doi:10.1016/j.jdermsci.2018.08.012.
6. Swanson, M.A.; Miller, K.; Young, S.P.; Tong, S.; Ghaloul-Gonzalez, L.; Neira-Fresneda, J.; Schlichting, L.; Peck, C.; Gabel, L.; Friederich, M.W.; et al. Cerebrospinal Fluid Amino Acids Glycine, Serine, and Threonine in Nonketotic Hyperglycinemia. *J. Inherit. Metab. Dis.* **2022**, *45*, 734–747, doi:10.1002/jimd.12500.
7. Razak, M.A.; Begum, P.S.; Viswanath, B.; Rajagopal, S. Multifarious Beneficial Effect of Nonessential Amino Acid, Glycine: A Review. *Oxid. Med. Cell. Longev.* **2017**, *2017*, 1716701, doi:10.1155/2017/1716701.
8. Ecker, K.; Hengst, L. Skp2: Caught in the Akt. *Nat. Cell Biol.* **2009**, *11*, 377–379, doi:10.1038/ncb0409-377.
9. Ibba, M.; Söll, D. Aminoacyl-TRNA Synthesis. *Annu. Rev. Biochem.* **2000**, *69*, 617–650, doi:10.1146/annurev.biochem.69.1.617.
10. Goodarzi, H.; Nguyen, H.C.B.; Zhang, S.; Dill, B.D.; Molina, H.; Tavazoie, S.F. Modulated Expression of Specific TRNAs Drives Gene Expression and Cancer Progression. *Cell* **2016**, *165*, 1416–1427, doi:10.1016/j.cell.2016.05.046.
11. Torres, A.G.; Batlle, E.; Ribas de Pouplana, L. Role of TRNA Modifications in Human Diseases. *Trends Mol. Med.* **2014**, *20*, 306–314, doi:10.1016/j.molmed.2014.01.008.
12. Jones, O.A.; Maguire, M.L.; Griffin, J.L. Environmental Pollution and Diabetes: A Neglected Association. *The Lancet* **2008**, *371*, 287–288, doi:10.1016/S0140-6736(08)60147-6.
13. Akifumi, E.; Hidenobu, M.; Chisato, M. The Effects of Early Postnatal Exposure to a Low Dose of Decabromodiphenyl Ether (BDE-209) on Serum Metabolites in Male Mice. *J. Toxicol. Sci.* **2016**, *41*, 667–675, doi:10.2131/jts.41.667.
14. Gray, L.R.; Tompkins, S.C.; Taylor, E.B. Regulation of Pyruvate Metabolism and Human Disease. *Cell. Mol. Life Sci.* **2014**, *71*, 2577–2604, doi:10.1007/s00018-013-1539-2.
15. Kunze, M.; Hartig, A. Permeability of the Peroxisomal Membrane: Lessons from the Glyoxylate Cycle. *Front. Physiol.* **2013**, *4*, doi:10.3389/fphys.2013.00204.
16. Li, J.; Hu, Y.; Liu, L.; Wang, Q.; Zeng, J.; Chen, C. PM2.5 Exposure Perturbs Lung Microbiome and Its Metabolic Profile in Mice. *Sci. Total Environ.* **2020**, *721*, 137432, doi:10.1016/j.scitotenv.2020.137432.
17. Almannai, M.; Wang, J.; Dai, H.; El-Hattab, A.W.; Faqeih, E.A.; Saleh, M.A.; Al Asmari, A.; Alwadei, A.H.; Aljadhai, Y.I.; AlHashem, A.; et al. FARS2 Deficiency; New Cases, Review of Clinical, Biochemical, and Molecular Spectra, and Variants Interpretation Based on Structural, Functional, and Evolutionary Significance. *Mol. Genet. Metab.* **2018**, *125*, 281–291, doi:10.1016/j.ymgme.2018.07.014.
18. Cao, T.; Gao, Z.; Gu, L.; Chen, M.; Yang, B.; Cao, K.; Huang, H.; Li, M. AdipoR1/APPL1 Potentiates the Protective Effects of Globular Adiponectin on Angiotensin II-Induced Cardiac

Hypertrophy and Fibrosis in Neonatal Rat Atrial Myocytes and Fibroblasts. *PLOS ONE* **2014**, *9*, e103793, doi:10.1371/journal.pone.0103793.
19. Liu, R.; Meng, J.; Lou, D. Adiponectin Inhibits D-gal-induced Cardiomyocyte Senescence via AdipoR1/APPL1. *Mol. Med. Rep.* **2021**, *24*, 1–10, doi:10.3892/mmr.2021.12358.
20. Yamano, K.; Youle, R.J. Two Different Axes CALCOCO2-RB1CC1 and OPTN-ATG9A Initiate PRKN-Mediated Mitophagy. *Autophagy* **2020**, *16*, 2105–2107, doi:10.1080/15548627.2020.1815457.
21. Zhang, C.; Nie, P.; Zhou, C.; Hu, Y.; Duan, S.; Gu, M.; Jiang, D.; Wang, Y.; Deng, Z.; Chen, J.; et al. Oxidative Stress-Induced Mitophagy Is Suppressed by the MiR-106b-93-25 Cluster in a Protective Manner. *Cell Death Dis.* **2021**, *12*, 209, doi:10.1038/s41419-021-03484-3.
22. Yang, C.; Rodionov, D.A.; Rodionova, I.A.; Li, X.; Osterman, A.L. Glycerate 2-Kinase of Thermotoga Maritima and Genomic Reconstruction of Related Metabolic Pathways. *J. Bacteriol.* **2008**, *190*, 1773–1782, doi:10.1128/JB.01469-07.
23. de Freitas-Silva, L.; Rodríguez-Ruiz, M.; Houmani, H.; da Silva, L.C.; Palma, J.M.; Corpas, F.J. Glyphosate-Induced Oxidative Stress in Arabidopsis Thaliana Affecting Peroxisomal Metabolism and Triggers Activity in the Oxidative Phase of the Pentose Phosphate Pathway (OxPPP) Involved in NADPH Generation. *J. Plant Physiol.* **2017**, *218*, 196–205, doi:10.1016/j.jplph.2017.08.007.
24. Li, X.; Schimenti, J.C. Mouse Pachytene Checkpoint 2 (Trip13) Is Required for Completing Meiotic Recombination but Not Synapsis. *PLOS Genet.* **2007**, *3*, e130, doi:10.1371/journal.pgen.0030130.
25. Clairmont, C.S.; Sarangi, P.; Ponnienselvan, K.; Galli, L.D.; Csete, I.; Moreau, L.; Adelmant, G.; Chowdhury, D.; Marto, J.A.; D'Andrea, A.D. TRIP13 Regulates DNA Repair Pathway Choice through REV7 Conformational Change. *Nat. Cell Biol.* **2020**, *22*, 87–96, doi:10.1038/s41556-019-0442-y.
26. Yuan, Q.; Tang, B.; Zhang, C. Signaling Pathways of Chronic Kidney Diseases, Implications for Therapeutics. *Signal Transduct. Target. Ther.* **2022**, *7*, 182, doi:10.1038/s41392-022-01036-5.
27. Park, S.; Kim, S.; Kim, M.J.; Hong, Y.; Lee, A.Y.; Lee, H.; Tran, Q.; Kim, M.; Cho, H.; Park, J.; et al. GOLGA2 Loss Causes Fibrosis with Autophagy in the Mouse Lung and Liver. *Biochem. Biophys. Res. Commun.* **2018**, *495*, 594–600, doi:10.1016/j.bbrc.2017.11.049.
28. Kubota, Y.; Nomura, K.; Katoh, Y.; Yamashita, R.; Kaneko, K.; Furuyama, K. Novel Mechanisms for Heme-Dependent Degradation of ALAS1 Protein as a Component of Negative Feedback Regulation of Heme Biosynthesis. *J. Biol. Chem.* **2016**, *291*, 20516–20529, doi:10.1074/jbc.M116.719161.
29. Niittynen, M.; Tuomisto, J.T.; Pohjanvirta, R. Effect of 2,3,7,8-Tetrachlorodibenzo-p-Dioxin (TCDD) on Heme Oxygenase-1, Biliverdin IXalpha Reductase and Delta-Aminolevulinic Acid Synthetase 1 in Rats with Wild-Type or Variant AH Receptor. *Toxicology* **2008**, *250*, 132–142, doi:10.1016/j.tox.2008.06.014.
30. Papaconstantinou, J. The Role of Signaling Pathways of Inflammation and Oxidative Stress in Development of Senescence and Aging Phenotypes in Cardiovascular Disease. *Cells* **2019**, *8*, 1383, doi:10.3390/cells8111383.
31. Xie, X.; Deng, T.; Duan, J.; Xie, J.; Yuan, J.; Chen, M. Exposure to Polystyrene Microplastics Causes Reproductive Toxicity through Oxidative Stress and Activation of the P38 MAPK Signaling Pathway. *Ecotoxicol. Environ. Saf.* **2020**, *190*, 110133, doi:10.1016/j.ecoenv.2019.110133.
32. Newey, P.J.; Bowl, M.R.; Thakker, R.V. Parafibromin--Functional Insights. *J. Intern. Med.* **2009**, *266*, 84–98, doi:10.1111/j.1365-2796.2009.02107.x.
33. Deska, M.; Romuk, E.; Segiet, O.; Polczyk, J.; Buła, G.; Gawrychowski, J. Oxidative Stress in Proliferative Lesions of Parathyroid Gland. *Pol. Przegl. Chir.* **2018**, *91*, 29–34, doi:10.5604/01.3001.0012.7258.
34. Moulin, P.; Vaysse, F.; Bieth, E.; Mornet, E.; Gennero, I.; Dalicieux-Laurencin, S.; Baunin, C.; Tauber, M.T.; De Gauzy, J.S.; Salles, J.P. Hypophosphatasia May Lead to Bone Fragility: Don't Miss It. *Eur. J. Pediatr.* **2009**, *168*, 783–788, doi:10.1007/s00431-008-0835-6.
35. Tsaprouni, L.G.; Yang, T.-P.; Bell, J.; Dick, K.J.; Kanoni, S.; Nisbet, J.; Viñuela, A.; Grundberg, E.; Nelson, C.P.; Meduri, E.; et al. Cigarette Smoking Reduces DNA Methylation Levels at Multiple Genomic Loci but the Effect Is Partially Reversible upon Cessation. *Epigenetics* **2014**, *9*, 1382–1396, doi:10.4161/15592294.2014.969637.

36. Wu, D.-D.; Irwin, D.M. Evolution of Trichocyte Keratin Associated Proteins. In *The Hair Fibre: Proteins, Structure and Development*; Plowman, J.E., Harland, D.P., Deb-Choudhury, S., Eds.; Advances in Experimental Medicine and Biology; Springer: Singapore, 2018; pp. 47–56 ISBN 978-981-10-8195-8.
37. Si, M.; Lang, J. The Roles of Metallothioneins in Carcinogenesis. *J. Hematol. Oncol.J Hematol Oncol* **2018**, *11*, 107, doi:10.1186/s13045-018-0645-x.
38. Baszuk, P.; Janasik, B.; Pietrzak, S.; Marciniak, W.; Reszka, E.; Białkowska, K.; Jabłońska, E.; Muszyńska, M.; Lesicka, M.; Derkacz, R.; et al. Lung Cancer Occurrence-Correlation with Serum Chromium Levels and Genotypes. *Biol. Trace Elem. Res.* **2021**, *199*, 1228–1236, doi:10.1007/s12011-020-02240-6.
39. Williams, E.L.; Acquaviva, C.; Amoroso, A.; Chevalier, F.; Coulter-Mackie, M.; Monico, C.G.; Giachino, D.; Owen, T.; Robbiano, A.; Salido, E.; et al. Primary Hyperoxaluria Type 1: Update and Additional Mutation Analysis of the AGXT Gene. *Hum. Mutat.* **2009**, *30*, 910–917, doi:10.1002/humu.21021.
40. Apanasets, O.; Grou, C.P.; Van Veldhoven, P.P.; Brees, C.; Wang, B.; Nordgren, M.; Dodt, G.; Azevedo, J.E.; Fransen, M. PEX5, the Shuttling Import Receptor for Peroxisomal Matrix Proteins, Is a Redox-Sensitive Protein. *Traffic* **2014**, *15*, 94–103, doi:10.1111/tra.12129.
41. Buday, K.; Conrad, M. Emerging Roles for Non-Selenium Containing ER-Resident Glutathione Peroxidases in Cell Signaling and Disease. *Biol. Chem.* **2021**, *402*, 271–287, doi:10.1515/hsz-2020-0286.
42. Chen, Y.-I.; Wei, P.-C.; Hsu, J.-L.; Su, F.-Y.; Lee, W.-H. NPGPx (GPx7): A Novel Oxidative Stress Sensor/Transmitter with Multiple Roles in Redox Homeostasis. *Am. J. Transl. Res.* **2016**, *8*, 1626–1640.
